# Capturing the perfect pose

**DOI:** 10.1038/s42003-021-02591-3

**Published:** 2021-09-09

**Authors:** Anam Akhtar

**Affiliations:** Communications Biology, https://www.nature.com/commsbio

## Abstract

Lipid nanoparticles can be used to deliver nucleic acids for gene expression modulation—but getting them to target specific tissues is an ongoing challenge. In a new study by Dammes et al., a conformation-sensitive targeting strategy is used to achieve better selectivity in silencing gut-homing leukocytes in mouse models of colitis.

The intestinal immune system is continuously faced with a very delicate task: to mount an offense against pathogenic bacteria, while maintaining tolerance towards good bacteria living in the gut. When this fine balance is disrupted, aberrant inflammatory responses cause chronic intestinal inflammation, such as inflammatory bowel disease (IBD)^1^. There is no satisfactory cure for IBD yet and blocking cytokines or receptors with antibodies provide only temporary relief. One treatment strategy, which has shown promise, is to change the behaviour of inflammatory leukocytes by rewiring their gene expression. This tricky task can be achieved by delivering nucleic acids capable of silencing gene expression to the affected cells. This fragile cargo (nucleic acids) can be transported to its destination and kept safe from the hostile in vivo environment with lipid nanoparticles (LNPs), frequently used as delivery vehicles. LNPs are especially good candidates with high encapsulation efficiency, low batch-to-batch variation and tendency to easily fuse in membranes. However, LNPs come with one big caveat. After intravenous injection, they have a tendency to travel straight to the liver and accumulate, making it difficult to direct them to other cell types. Arming these LNPs with additional homing devices can increase their selectivity in reaching the target cells, as shown in a recent study by Dan Peer and colleagues at Harvard Medical School^2^.

Since IFN-γ is known to be causatively involved in colitis, one of the two main forms of IBD, the gene encoding this protein (*Ifng*) was chosen as the target for gene silencing. Once the target and vehicle were decided, the next important step was to find a homing device that could be used to successfully target the LNPs to the correct inflammatory leukocytes. In IBD, one of the key proteins attracting leukocytes to the gut during intestinal inflammation is α_4_β_7_ integrin. Even though this protein is abundant in the intestinal T-cell population and in circulating CD4+ T cells of healthy individuals, it differentiates itself in the diseased state by adopting a specific, high-affinity conformation when activated. The authors cleverly devised a strategy to only target this particular conformation, and consequently increase the specificity of their targeting (Fig. 1). Hence, the mucosal addressin cell adhesion molecule-1 (MAdCAM-1) molecule, which specifically binds to the high-affinity conformation of α_4_β_7_ integrin could be the much-needed homing device to their LNP vehicle.

**Fig. 1:**
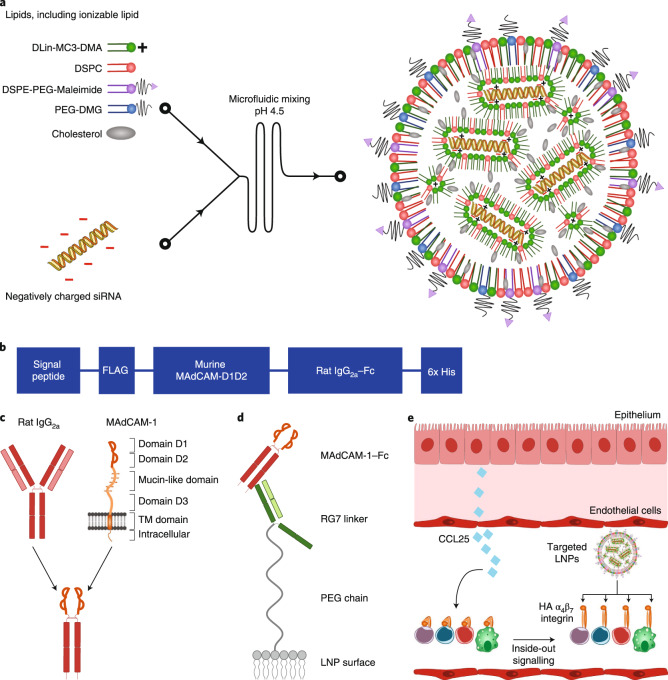
LNPs design to target high affinity conformation of α4β7 integrin. **a** Illustration of the generation of LNPs using microfluidics. The ionizable lipid facilitates short interfering RNA (siRNA) encapsulation through its positive charge at low pH. **b** Overview of the different domains of the mucosal vascular addressin cell adhesion molecule-1 (MAdCAM-1)–Fc fusion protein. **c** Overview of the fusion strategy. The different domains of the wild-type MAdCAM-1 are shown. Only the integrin-binding domains D1 and D2 are used. D1D2 is fused to the hinge of rat IgG2a with a flexible linker. **d** Schematic drawing depicting the conjugation strategy of the MAdCAM-1–Fc to the LNPs. The RG7 linker (mAb against rat IgG2a) is chemically conjugated with the LNPs to the maleimide group in the lipid DSPE-PEG-maleimide. RG7 readily binds the MAdCAM-1–Fc by antibody affinity. **e** LNP targeting to HA α4β7 integrin. CCL25 induces the integrin conformational change. Reprinted by permission from Springer Nature Customer Service Centre GmbH: Springer Nature, *Nature Nanotechnology*, from Conformation-sensitive targeting of lipid nanoparticles for RNA therapeutics, Dammes, N., Goldsmith, M., Ramishetti, S. et al., © 2021.

Indeed, the danger associated with insufficient specificity when blocking leukocyte homing is brought into sharp relief by the case of natalizumab, conformation-insensitive monoclonal antibodies against α4 integrin, which was temporarily withdrawn from the market for the treatment of IBD, since it increased the risk of severe infection, due to sequestering immune cells^3^. The modified LNPs that specifically target α_4_β_7_^+^ integrin-expressing cells in a conformation-dependent manner could be a potential solution to the above grim scenario.

Dan Peer and colleagues began by making a fusion recombinant protein that contained two integrin-binding domains from MAdCAM-1 linked it to LNP using a monoclonal secondary antibody, referred to as RG7. This linker binds MAdCAM-1 protein in such a way that leaves its integrin-binding domain free. Before testing gene silencing in the mouse model of colitis, the authors first went on to confirm the safety of LNPs as delivery vehicles. Indeed, injection of the LNPs did not cause any observable liver toxicity or induce unwanted immune responses. When mice were treated with MAdCAM-1 domain linked LNPs loaded with siIFN-γ, a strong therapeutic response in mice was observed compared to the controls.

Given the chronic nature of IBD and the life-long dependence on medicines for it, non-specific treatments can potentially lead to global immune suppression and sensitise a patient to opportunistic infections. The strategy employed in this study is therefore very smart, since it specifically captures one pose of a protein and thereby improves the specificity of the treatment. Since the relationship between conformational state and protein functionality is a well-known phenomenon, targeting a specific conformation of proteins will open new avenues in precise treatment strategies. The authors have shown us the first step that conformational changes of integrin can be exploited to enhance specificity and cellular uptake, which can translate into therapeutic efficacy in experimental colitis. However, there are still several unanswered questions which might challenge the clinical relevance of this approach, including what percentage of leukocytes assume that conformation, at which location and for how long? What other factors are involved in these chemokine-driven signalling changes which ultimately lead to such conformational changes and, also, can the design of their LNP be improved by replacing the possibly immunogenic rat IgG linker with a more suitable one? These questions aside, there is no doubt that this study presents an exciting approach to developing more specific drug delivery systems by exploiting a very basic feature of protein targeting: their conformational changes.
